# Mosquitoes and transmission of malaria parasites – not just vectors

**DOI:** 10.1186/1475-2875-3-39

**Published:** 2004-11-08

**Authors:** Richard EL Paul, Mawlouth Diallo, Paul T Brey

**Affiliations:** 1 Unité de Biochimie et Biologie Moléculaire des Insectes, Institut Pasteur, 28 rue du Dr. Roux, 75724 Paris cedex 15, France; 2 Laboratoire d'Entomologie Médicale, Institut Pasteur de Dakar, 36, Avenue Pasteur BP 220; Dakar, Sénégal

## Abstract

The regional malaria epidemics of the early 1900s provided the basis for much of our current understanding of malaria epidemiology. Colonel Gill, an eminent malariologist of that time, suggested that the explosive nature of the regional epidemics was due to a sudden increased infectiousness of the adult population. His pertinent observations underlying this suggestion have, however, gone unheeded. Here, the literature on *Plasmodium *seasonal behaviour is reviewed and three historical data sets, concerning seasonal transmission of *Plasmodium falciparum*, are examined. It is proposed that the dramatic seasonal increase in the density of uninfected mosquito bites results in an increased infectiousness of the human reservoir of infection and, therefore, plays a key role in "kick-starting" malaria parasite transmission.

## Introduction

A strategic aim of the global commitment to Roll Back Malaria is the development of reliable and practicable forecasting methods to enable the containment of epidemics. Much of the basis of our knowledge about the causes and dynamics of malaria epidemics comes from the qualitative analyses of the large scale, regional epidemics occurring in the first third of the last century [1-3]. Such regional epidemics have been consistently associated with unusual seasonal climatic conditions, namely those favourable for malaria transmission, but which follow years of unfavourable conditions. Favourable conditions are those that promote both anopheline density and longevity, notably high atmospheric humidity [1]. Thus, epidemics occur when there are "unusually" favourable transmission conditions in an otherwise poorly immune population and that an epidemic reflects the loss of equilibrium between the degree of immunity and the force of infection [1,2]. The implementation of remote sensing technologies for use in early warning systems for predicting epidemics have been developed on the basis of these original observations [4] and the development of more precise measurement of key parameters, such as temperature and rainfall anomaly, is in progress. However, as pointed out, the application of such new technologies must be carried out within the context of the wealth of knowledge concerning the basic epidemiological processes of malaria [5]. In this spirit, recent advances in mathematical epidemiology have paid special interest to the formal analysis of the cyclical nature of epidemics and how the epidemiological system responds to demographic and environmental variability [4-8]. One conclusion from such analyses is that a deeper comprehension of longitudinal epidemiological patterns requires a more detailed appreciation of the intra-host parasite population dynamics, including the effects of co-infection with both multiple strains and species of *Plasmodium *[9].

Despite the extension by Macdonald of the basic models of malaria, pioneered by Ross and Lotka, to introduce some level of biological complexity, many points raised at the time of the great regional epidemics remain unexplored or attributed to the error inherent in estimating biological parameters [10]. In his reappraisal of epidemics in general [1] and of the 1934–5 Ceylon epidemic in particular [11], Gill not only emphasised the value of climatic features in predicting epidemics, but also made several observations that ran contrary to the opinion of malariologists of the day. Firstly, he noted that during the first five weeks of the epidemic, there was a complete absence of child morbidity. He subsequently suggested that the explosive nature of the Ceylon epidemic must have been due to a sudden increase in the infectivity of the adult population, and that, therefore, the beginning of the epidemic was characterised by relapses in the adult population that then generated the necessary human transmission population [11,12]. This was countered at the time by malariologists who believed, based on fever charts and the absence of gametocytes, that the initial wave of morbidity was due to novel infections [13]. However, to date there has been no satisfactory explanation to account for the age-structured nature of the morbidity rise, other than relapses (of both *Plasmodium vivax *and *Plasmodium falciparum*) in the adult population. If Gill was indeed correct, then what caused the sudden increase in infectiousness of the adult population?

Seasonal epidemic rises superimposed on endemic prevalence are probably characteristic of most regions with endemic malaria [2]; there is always a degree of seasonality in mosquito bionomics. If the unusually favourable conditions that generate epidemics are an extreme example of more common seasonal variations in transmission intensity, Gill's pertinent observations may throw light on a fundamental question in malaria epidemiology: How exactly does transmission restart upon return of the mosquitoes. Current wisdom suggests that the sheer number of mosquitoes under favourable conditions results in the rapid expansion of the parasite population from a few initial source infections – as formalized in the classic Ross-Macdonald model of malaria, *Plasmodium *(*falciparum*) has a very high R_0 _(reproductive rate) that is strongly dependent on the mosquito biting rate. But where do the initial source infections come from? Several authors have noted the absence of gametocytes during inter-epidemic periods, but commented that a geometric rise in gametocyte carriers could generate the observed rapid increase in gametocyte rates during the course of an epidemic [14-16]. In an endemic field situation, chronic infections were found to produce gametocytes throughout the dry season in Sudan, although the transmissibility of such gametocytes was not assessed [17,18]. However, induced infection studies in naive individuals have shown that a *P. falciparum *infection can produce infective gametocytes throughout its infection duration [19]. Even this, however, cannot explain the explosive nature of the Ceylon epidemic [11], which strongly suggests that there may be underlying seasonal changes in intra-host parasite population dynamics that could have an effect on parasite transmission to mosquitoes. Inherent seasonality in some *Plasmodium *spp. (notably *P. vivax*), is well known and yet rarely considered in models of malaria epidemiology. In this paper, the nature of seasonality in malaria parasites is addressed and by examination of three historical data sets from Africa and Asia, it is proposed that uninfected mosquito bites play a significant role in increasing the infectiousness of the human reservoir of infection to mosquitoes. Thus, the intense expansion of the mosquito population at the start of the seasonal transmission season (whether resulting in an epidemic or not) has a significant biological effect above and beyond the role of vector.

### Seasonal chronology of infections: relapses, recrudescences and gametocyte production

#### (a) Temperate regions

The vernal (late winter – early spring) rise in the incidence of *P. vivax *in temperate regions is legendary, and was discussed in depth by Swellengrebel and De Buck (1938) [20]. Such vernal fevers occur in the apparent absence of *Anopheles*. Korteweg proposed that such fevers were the result of infections incubating from the previous year. Induced infection studies, implementing *P. vivax *and *P. falciparum *to treat neurosyphylitic patients, by Swellengrebel and others confirmed Korteweg's hypothesis that vernal fevers were due (at least in part) to latent vivax infections from the previous autumn [20-22]. A variety of strains were used in the course of such treatment, leading to the conclusion that there are inherent differences in the seasonal behaviour of strains, depending on their geographical (latitudinal) origin. James (1931) [21], using the Madagascar strain, found that only 1.6% of the infections had extended incubation periods (200–317 days); similarly Boyd & Kitchen (1949) [22], using the McCoy (southern USA) strain, found only 1.1% with incubation periods in excess of 75 days. In contrast, Swellengrebel and de Buck (1938) [20], using the Dutch strain, found 38% with protracted incubation periods. Thus, temperate strains were considered to exhibit inherent seasonal behaviour, emerging when the insect vector commences its seasonal activity.

Transmission to mosquitoes is achieved through the production of gametocytes. Ziemann (1914) [23] noted that a feature of the *P. vivax *spring relapses was a tendency for gametocyte production just before mosquitoes appeared. This is a feature of other haemosporidian parasites, most notably *Leucocytozoan *and *Haemoproteus *spp. that are widespread parasites of birds and lizards, the latter of which have recently been shown to be paraphyletic with *Plasmodium *species [24]. These parasites differ from *Plasmodium *spp. in maintaining the infection exclusively by exo-erythrocytic forms and only gametocytes are present in the circulating blood system; seasonal activity and the timing of gametocyte production are thus simultaneous in these genera. There is a rapid appearance of gametocytes in March and April, which is generally too early for novel infections by simulid black flies or ceratopogonid midges [25]; rather infection relapse appears to be related to altering hormone levels prior to the bird's reproductive season. *Haemoproteus *infections normally relapse during host breeding, peaking during egg laying and decreasing throughout the nestling rearing phase [26]. This reduction is attributable to an increase in immune function accompanied by a down-regulation of hormone levels [27]. An influence of sex hormones on gametocyte production is also suspected for *Plasmodium *infections in lizard malaria species. Gametocyte production is intensified during mid-late summer in *Plasmodium mexicanum *infections in lizards when similar seasonal fluctuations occur in testosterone levels. Although testosterone does not seem to affect overall parasite density and the course of infection, elevated testosterone significantly reduced variation in the timing of the onset of gametocyte production [28].

#### (b) Tropical regions and *P. falciparum*

In contrast to temperate and subtropical zones, seasonal patterns of parasite relapse in parasites such as *P. vivax *and *Hepatocystis *spp. (tropical relatives of *Haemoproteus *infecting baboons) in the tropics is less evident and thought not to occur [29]. Unlike temperate zones, seasons in the tropics are based largely on rainfall and are, therefore, intimately linked to mosquito bionomics. Given this intimate link between season, mosquitoes and transmission, how can seasonality in *P. falciparum *be detected? As stated previously, current wisdom holds that there is a continual production of low numbers of gametocytes that are the source of infection once mosquito numbers expand. However, contrary to accepted belief, mosquitoes can be found at all times of the year, even under very hostile climatic conditions [30] and seasonal mosquito activity is, therefore, primarily one of greatly increased numbers. Thus, as an initial test of transmission seasonality, the occurrence of infected "out of season" anophelines is examined. The traditional explanation for the absence of infections in anophelines is that climatic conditions reduce the lifespan of the adult mosquitoes such that the probability of their surviving long enough to allow completion of sporogonic development is negligible – that is, the vectorial capacity [31] is considerably reduced by the decreasing vector longevity. However, by examining the mosquito for oocyst (midgut) as well as sporozoite (salivary gland) infections, the extent of human infectiousness can be established irrespective of whether there is actual transmission. Bentley (1911) [32] summarizes several studies of this nature where anopheline stomachs and salivary glands were examined on a seasonal basis (Table 1). Although admittedly very limited, these data argue against a persistent level of human infectiousness to mosquitoes.

**Table 1:** Comparison of the oocyst and sporozoite rates in mosquitoes sampled during the non-transmission and transmission seasons. Data from Bentley (1911) [32].

Author	Place	Season	N° anophelines dissected for oocyst/sporozoites	Oocyst positive (%)	Sporozoite positive (%)
Bentley 1911	Bombay	Nov-June	178 / 123	0	0
		July-Oct	659 / 703	12.7	4.1
Gosio 1905	Tuscany	Apr-June	318	0	0
		July-Oct	512	27	4
Daniels	British Central Africa	Dry season	1500	0	0

Such anopheline data are one side of the coin and gametocytes are the other. If tropical seasons are defined most precisely by mosquito bionomics, it is conceivable that *Plasmodium *spp. would have evolved to respond to the mosquitoes themselves. Thus, in a manner akin to temperate parasite species that utilize vertebrate host seasonal cues to produce gametocytes at the optimal time, tropical species may respond to tropical cues – the mosquito bites. Is there any evidence that *P. falciparum*, for example, produces gametocytes in response to mosquito bites? As with *P. vivax*, *P. falciparum *gametocyte production has been found to peak at a time when the anopheles abundance was at a maximum [33-35]. However, this peak gametocyte rate occurred notably after the peak in the number of clinical cases and, thus, after the peak in transmission. Such an increase in gametocyte rate would be expected as a result of novel infections and, thus, characteristic of an endemic region during the transmission season. However, what evidence is there that, at the beginning of the transmission season, there is an increase in infectiousness of humans to mosquitoes that is not due to novel infections, but due to relapses in existing chronic infections. Identifying the human reservoir of infection and correlating gametocyte density with transmission success to mosquitoes is far from clear [36]. The human reservoir of infection (during the non-transmission season) will depend on the rate of recovery from infection, which in turn depends on the extent of previous exposure and the immune reaction to infection. Both these factors will alter with age for a given intensity of transmission. Therefore, in the absence of novel infections, the human reservoir of infection will have an age-specific distribution. If mosquito bites are having a gametocyte-promoting effect on existing chronic infections, then age-specific patterns of gametocyte production might be expected. However, although increasing gametocyte density tends to result in greater infectiousness to mosquitoes [19,37,38], it has been repeatedly demonstrated that high gametocyte densities do not guarantee high mosquito infection rates [19,38-40]. Cryptic infectors with no or very few apparent gametocytes, are capable of infecting mosquitoes and may contribute to a very significant proportion of the human transmission reservoir [39-41]. Moreover, gametocyte density varies greatly according to the region of study, and also with age (i.e. history of exposure) of the individual and tends to reflect the overall asexual parasite density [42,43]; consequently, infections in the younger and therefore less immune individuals tend to produce higher densities of gametocytes [44-46] than adults. Therefore gametocyte density itself may not be a sufficiently sensitive indicator. Rather, age-specific seasonal changes in intra-host parasite prevalence rates are preferred. This measure encompasses changes in both sexual and asexual prevalence rates and the tendency for an infection in a particular age group to produce gametocytes; it is, thus, a measure of parasite behaviour within a particular age-group during a certain season. To examine the potential role of mosquito bites on the seasonal nature of *P. falciparum *transmission, three historical data sets, addressing age-specific longitudinal patterns of *P. falciparum *asexual and sexual prevalence rates in relation to mosquito abundance patterns, are analyzed. One of the major shortcomings of these studies is that they did not have recourse to PCR (quantitative, reverse transcriptase) technology and thus will have underestimated parasite prevalence rates and can not distinguish a relapsed chronic infection from a novel infection. However, despite such limitations, the data sets reveal much on the seasonality of *P. falciparum *transmission.

### Study 1 : Barber & Olinger (Ref: 47.1931 – Urban, infant/mother, Lagos, Nigeria)

For a period of 18 months, records of vaccine visits to the Lagos local health office of 3–4 month old babies with their mothers provided *P. falciparum *seasonal prevalence data. During the same period, intensive surveys of anopheline activity and mosquito infection prevalence rate (both salivary gland sporozoite and midgut zygote (oocyst) infection rates) were carried out throughout Lagos by indoor resting catches in from 200 to more than 400 rooms. Infants (three to four months) are no longer expected to be protected by maternal antibodies and prevalence rates in this very young "naïve" age group provide a good indication of the force of infection and hence the current transmission intensity. Mothers will include a distribution of older age groups that are expected to have developed some immunity to infection and disease. It should be noted that although *Plasmodium *infection characteristics in pregnant and recently post-partum women differ from non-gravid women of similar ages [48,49], the mothers here are three to four months post-partum and so likely to be representative of the adult population. One confounding factor of the data set is that urban populations are mobile and individuals may thus have acquired infections elsewhere. Although it is likely that mothers and infants would have travelled together and thus be exposed to identical mosquito biting rates, the absence of paired mother-infant data weaken any comparison of parasite rates.

Using the mosquito data, the estimated monthly entomological inoculation rate (EIR) (mosquito density × mosquito biting rate × sporozoite rate) and the estimated human reservoir of infection (infectious gametocyte rate) are calculated, using the formula of Macdonald (1952) [50].

 where *s *(sporozoite rate),  (infective mosquito lifespan), *a *(human biting rate; here estimated as 0.33) and *x *is the proportion of human infections that are infectious to mosquitoes (hereon referred to as estimated gametocyte infectious rate. Note this is not necessarily equivalent to the gametocyte rate).

*p *can be calculated from the measured total mosquito infection rates: sporozoite rate, *s*, and zygote rate, *z*.



where *n *and *m *are the number of days required for the development of identifiable presence of sporozoites and oocysts respectively (i.e. the extrinsic incubation periods for sporozoites and oocysts). Here standard values of *n *= 12 and *m *= 3 [45,50] were taken using the Moshkovsky scale where  and a mean annual temperature *T *fluctuating around 25°C.

**Figure 1:**
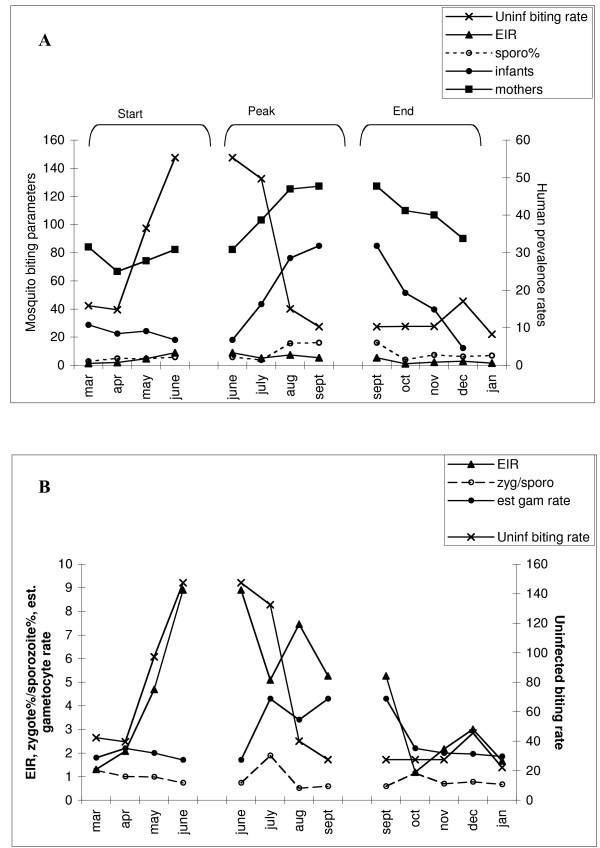
Mother-infant seasonal *P. falciparum *prevalence rates during routine vaccination visits. Data adapted from Barber & Olinger (1931) [47], Lagos, Nigeria. (a) *P. falciparum *prevalence rates in three month old infants and their mothers. Anopheline mosquito inoculation parameters from selected houses throughout Lagos include the Entomological Inoculation Rate (EIR), which is the number of infected mosquito bites per person per month; the Uninfected biting rate, which is the number of uninfected mosquito bites per person per month; the sporozoite rate, which is the percentage of mosquitoes with sporozoites in their salivary glands. (b) Anopheline biting rates (as above) and the relative proportion of mosquitoes with zygote (oocyst stage infections) compared with sporozoite stage infections from which the estimated gametocyte infectious rates can be calculated (see text).

In this way, values for *x *can be estimated, thereby enabling examination of how the infection rates in the two directions, mosquito  man (EIR) and man  mosquito (infectious gametocyte rates), relate to the age-specific (three to four month old infants versus mothers) seasonal changes in parasite prevalence rates and the mosquito activity patterns (Figs. 1a &1b).

### Interpretation

#### Start

At the beginning of the transmission season, there is a dramatic increase in anopheline biting density and, although the sporozoite rates do not increase, EIR increases from one to nine infectious bites per month (Fig. 1a). Paradoxically, however, although there is an increase in adult parasite prevalence rates, there is a decrease in infant prevalence rates (Fig. 1a). This latter, more sensitive marker of the force of infection, would suggest that any increase in transmission is offset by a greater infant recovery rate or that the increased EIR is not yet manifest in this age group. The estimated gametocyte infectious rate and the proportion of zygote vs. sporozoite infection prevalences decrease (Fig. 1b), further suggesting that the increase in EIR has little impact in generating novel or super infections that lead to gametocyte production.

#### Peak

By contrast, during the "Peak" of transmission, following the peak EIR (June), there is a parallel increase in prevalence of infection in both the young and older age groups (Fig. 1a), a large increase in both estimated gametocyte infectious rate and zygote/sporozoite proportions (Fig. 1b), prior to the decrease in mosquito numbers and longevity (August). Thus during this phase, the occurrence of novel/super infections is notable during the month after peak EIR (June) and is signalled not only by increases in prevalence of infection across all age groups (especially in the infant age class), but also by the increase in the estimated gametocyte infectious rate (Figs. 1a &1b). The EIR follows the sporozoite rate which increases in the absence of the emergence of new adult mosquitoes (note the drop in the mosquito activity).

#### End

In the final "End" phase, prevalence rates drop markedly and notably more rapidly in the younger age class (Fig. 1a), indicative of a greater rate of recovery from infection. The estimated gametocyte infectious rate drops, the zygote/sporozoite proportions return to an "equilibrium" value expected from the low and relatively stable mosquito densities. Transmission is, thus, minimal as this point, as indicated by the very low infant rates and the low estimated gametocyte infectious rate.

The data in the *Peak *and *End *phases follow the classic description of a malaria transmission season, most especially confirming the sensitivity of the infant versus the adult age groups in revealing changes in the transmission intensity and the relationship between transmission and the (estimated) patterns of gametocyte dynamics. However, the *Start *phase highlights a novel effect whereby there is little effective transmission (infant rates or estimated gametocyte rates) but an increase in adult prevalence rates, which notably parallels the dramatic increase in overall biting rate, predominantly by uninfected anophelines. This pattern suggests that the increase in mosquito activity *per se *may be having an effect on parasite prevalence rates in the adult population. In high endemicity areas, subpatent infections are common and likely to occur in older age groups with a previous history of exposure [51]. This contrasts with infants who respond to infections with strong fever and cytokine reactions, thus reducing the duration of infection. Such age-specific differences in immune response and duration of infections has been highlighted by molecular epidemiological studies of multiple clone infections by *P. falciparum *in highly endemic areas, which have demonstrated age dependence in both the multiplicity of infection and the relationships between this multiplicity and the risk of acute illness: in older children, a high multiplicity of infection is characteristic of low-level chronic parasitaemia [52,53]. In areas of high transmission, parasitaemias are likely to be determined mainly by the interaction of schizogony and anti-blood stage immunity, leading to periodic fluctuations in levels of parasitaemia [54]. Although the early increase in the parasite rates of the mothers in this reported study of Barber & Olinger (1931) [47] may be simply the result of stochastic variation in periodicity, the coherence of infant and mother parasite rates during the other phases does suggest that there may be a real biological cause underlying this apparently anomalous increase: that insect bites *per se *cause the re-emergence of existing infections in this adult population.

This hypothesis is to some extent corroborated by observed patterns of parasite prevalence versus parasite density in an adult population in Liberia [55]. Here, at the start of the transmission season, the prevalence of infection decreased but the mean parasite density increased. Rather than invoking superinfection, which would be at odds with the reduced prevalence rates, mosquito bites could actually be resulting in an increase in asexual (and thus sexual) parasite densities.

**Figure 2:**
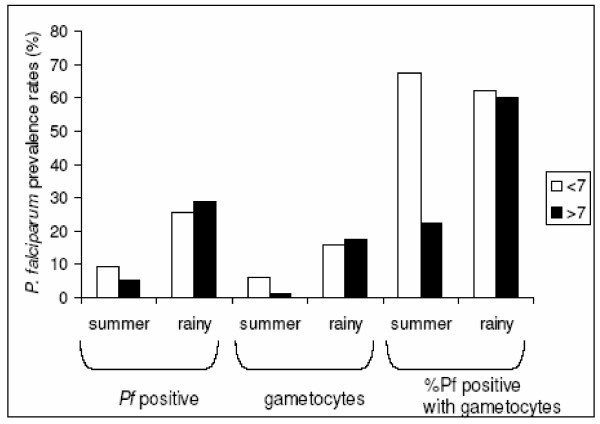
Age-specific (less than or greater than seven years old) seasonal *P. falciparum *all stage (*Pf *positive) prevalence rates; those with gametocytes and the proportion of *P. falciparum *positive individuals also with gametocytes. Data from Muirhead-Thomson, Jamaica (1952) [56].

Such age-specific effects have also been noted by Muirhead-Thomson [56] during longitudinal studies in Jamaica (Fig. 2). *Anopheles albimanus *productivity from larval collections was at a maximum at the very time when sudden cold spells were producing a wave of relapses and a crop of gametocyte carriers. At that critical period, at the beginning of the malaria season, the increase in gametocyte rate was particularly marked in the group >7 years of age. In this age group, the gametocyte rate increases from 1.1% in the summer to 17.3% in the rainy season. Muirhead-Thomson [57] suggested that these age groups were more often bitten and that therefore age-specific differences were simply a result of differential exposure; that is the older age group were subject to a higher force of infection. Although such an explanation is possible, the differences in the seasonal prevalence rates are negligible. By contrast, the relative differences in gametocyte rates in the young and older age groups are marked: in the younger age groups the proportion of *P. falciparum *infections positive for gametocytes varied little with season (from 67 to 62%), whereas in the older age group the change was marked, increasing from 22 to 61%. Why should the two groups display very different changes in gametocyte production but similar increases in prevalence rates? Could mosquito biting be having an effect on infections in the older age group?

### Study 2 : Wilson (Ref. 44.1936 – Rural, active case detection, Gombero, Tanzania)

**Figure 3:**
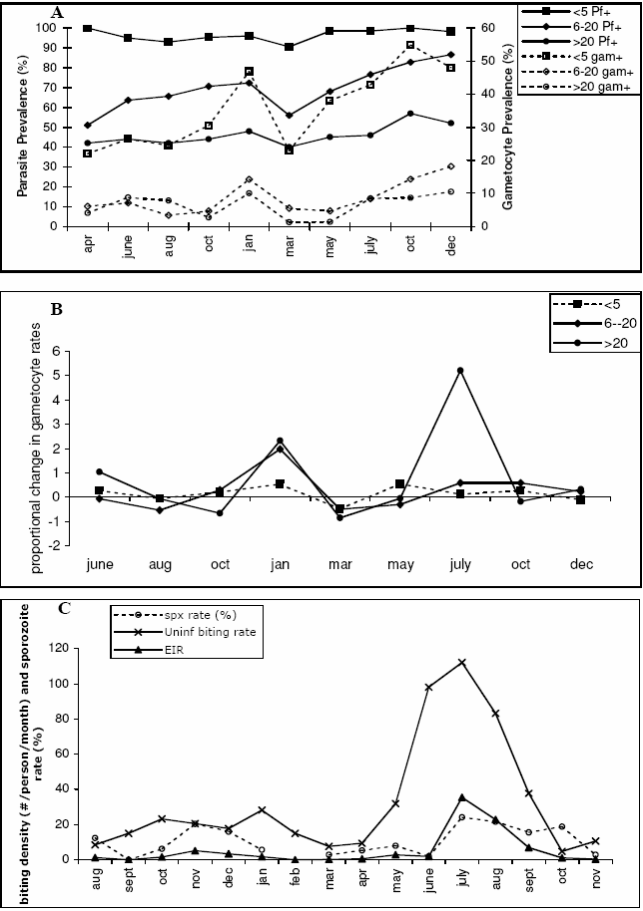
Seasonal study of *P. falciparum *prevalence rates in a rural village in Tanzania. Data from Wilson (1936) [44]. (a) Age-specific parasite prevalence rates (all stages and those with gametocytes). Three age groups are considered: less than five, from six to 20 and greater than 20 years old. (b) Age-specific rate of change in the proportion of infected individuals that also have gametocytes (i.e. "(Proportion gametocyte positive at month 'm+1'/proportion at month 'm') - 1)". A value of 0, therefore, means no relative change in the proportion of infected individuals with gametocytes. (c) Entomological parameters measured in selected houses. Number of infected (EIR) and uninfected mosquito bites per person per month and the sporozoite rate.

The second data set comes from a longitudinal study in a rural village in Tanzania characterised by seasonally intense transmission. In this study both gametocyte and asexual prevalence rates were measured intermittently (every two or three months) and classified into three age groups : <5 years, 6–20 years and 20+ years. In addition, anopheline activity and sporozoite rates were measured on a monthly basis. Wilson noted distinct seasonal fluctuations in gametocyte rates, whereas overall parasite prevalence rates varied little. The longitudinal age-specific parasite and gametocyte prevalence rates are shown in Fig. 3a. How the proportion of parasite positive individuals that also have gametocytes changes over time and by age group is shown in Fig. 3b. This is calculated simply as the proportion at time t+1 divided by the proportion at time t (1 is then subtracted from this figure such that a value of 0 indicates no change). The mosquito activity patterns and sporozoite rates are given in Fig. 3c.

### Interpretation

The parasite prevalence rates change very little but do generally follow the monthly fluctuations in EIR; the absence of monthly data points, however, abnegates any rigorous comparison. The age-specific prevalence rates are characteristic of hyperendemic transmission intensity with peak rates in the very youngest age group. As discussed in the previous study [47], the occurrence of asymptomatic chronic infections would be expected to increase with age. Despite missing monthly data points for the human parasite prevalence data, comparison of the three graphs highlights several distinct points. Most notably, although overall gametocyte rates increase most significantly in the infant age group, the proportional changes are most significant in the older age groups (January) and notably in the adult age group (July). The January rise in gametocyte rates follows the October – December increase in EIR and thus could be indicative of novel/super infections. However, the prevalence rates scarcely change in any age group. Although prevalence rate is considered an insensitive marker of alterations in transmission intensity, here they do clearly decrease in March following the absence of transmission in February and markedly increase during the unusually sustained transmission season from March to October. Superimposed on this sustained seasonal transmission is a significant increase in the gametocyte production rate in the adult population (July). As shown in Figs. 3a,3b,3c, this coincides with large increases in mosquito abundance (June & July). Several features argue against this being due to novel/super infections : (i) the age-specific nature of this increase, (ii) the absence of significant EIR in June and (iii) the absence of any additional increase in adult prevalence rates over and above that following the general trend from March to October. By contrast, following this gametocyte rise in July, there is an increase in sporozoite rates and hence the EIR and subsequent increases in overall prevalence rates in October, i.e. the July gametocyte rise provides the source of infection. As observed in the previous study, there appears to be a distinct effect of mosquito biting *per se *on parasite prevalence rates in the adult population.

### Study 3 : Rosenberg (Refs. 58–60 1990 – Rural, Active case detection, Thailand)

Rosenberg *et al*. (1990) [58-60] conducted a two year longitudinal study in a rural farming village with hyperendemic *P. falciparum *and *P. vivax *malaria in S.E. Thailand. During the two year study period, monthly human and mosquito data were collected. Despite the very different nature of malaria epidemiology in Thailand, when compared with the previous two African studies, Rosenberg *et al*. (1990) [60] noted very similar seasonal, mosquito associated fluctuations in gametocyte density to those observed in the Wilson study [44] – « Nonetheless, the pattern we observed in Thailand was strikingly similar to that which Wilson (1936) described for Tanzania 50 years earlier; the similarity is all the more remarkable as the 2 sites have virtually no features in common other than seasonally intense transmission. » Rosenberg *et al*. (1990) [60] discussed at length the seasonal fluctuations in gametocyte prevalence rates and noted that the perennial cycle of malaria incidence was more evident in the high trophozoite densities and the gametocyte prevalence rates than the gross prevalence rates. Such parasite fluctuations were interpreted as being the result of superinfections in 50% of the cases. The remaining 50% were considered to be the result of novel infections. However, they stated their uncertainty as to why such a considerable incidence of novel infections « ......did not inflate prevalence soon after transmission started » [60]. The pertinent data are displayed in Figs. 6a-c with the addition of the uninfected mosquito biting density [59]. In addition, from Rosenberg *et al*. [60] Table 1 and text, the following supplementary details are given: (i) 14+ age group harboured 49% of the gametocytes but 84.6% high gametocyte densities (<20/500 white blood cells) occurred in the group <14 years of age; (ii) dry season gametocyte prevalence was 2.6x higher than the wet season prevalence in <14 age group *vs*. 8x higher in the 14+ age group ; (iii) in year two, the EIR decreased by 67% and, whereas the gametocyte prevalence decreased in <14 age group, it increased in the 14+ group.

### Interpretation

The younger age groups have higher gametocyte densities reflecting their generally higher parasite loads. Younger age groups also tend to have higher rates of acquisition and recovery (i.e. turnover) and, therefore, most gametocytes would be expected to be due to novel infections. This is supported by the finding that gametocyte prevalence decreased in the <14 group in year 2 when inoculation rate and force of infection was less. Gametocyte prevalence increased in the 14+ group despite a decrease in inoculation rate in year two; note, however, that despite the drop in EIR, the mosquito biting intensity was higher. Thus, the apparent paradox of there being considerable novel infections at the beginning of the transmission season, but with no influence on parasite prevalence, may be explained by the re-emergence of pre-existing chronic infections in the 14+ age group. This group is more likely to harbour such infections and is shown here to display the most significant seasonal changes in gametocyte rates [60].

The sequence of events in this study could thus be interpreted as follows : In year one, the mosquito biting rate increases dramatically in October, with a concomitant monthly rise in oocyst rates, but not sporozoites (data not shown here), and no rise in gametocyte rates. In November, the sporozoite rate and EIR increase as expected from the previous month's oocyst rates and the decrease in newly emerged mosquitoes (evident from the parous rate [59]). Despite the absence of high *P. falciparum *trophozoite density infections, suggesting an absence of novel infections, there is a small rise in gametocyte rates. The following month (December), this increased EIR leads to a dramatic increase in high *P. falciparum *trophozoite density infections and gametocyte prevalence rates. A similar pattern is seen in the second year, and notably, although the EIR and sporozoite rates were lower, resulting in reduced gametocyte prevalence rates in children, the peak mosquito biting density (which again preceded the increase in sporozoite rate) was higher, as was the gametocyte rate in adults. Whereas the large concomitant increases in high *P. falciparum *trophozoite density infections and gametocyte prevalence rates can be taken as evidence for novel/super infections, the increase in gametocyte rates in the absence of sporozoites at the start of transmission, coupled with the variation in age-specific gametocyte rates between the two years, suggests once more that mosquito biting itself may induce gametocyte production and subsequently augment mosquito infection rates.

## Conclusions

Here, a case is presented for there being a role of uninfected mosquito bites in increasing the human reservoir of infection at the beginning of the transmission season. The available data is limited, but consistently suggest that the traditonal view that there is a rapid expansion of the parasite population from a few source infections following the seasonal increase in anophelines is overly simplistic. It must, however, be emphasised that in no way is it suggested that once transmission is under way, the epidemiology of malaria is not satisfactorily described by classic Ross-Macdonald models or variants thereof [10]. Rather, it is simply proposed that the image of intense parasite activity and dynamics during peak transmission detracts from a more subtle process occurring at the very beginning of the season. That is, Gill [11,12] may have been correct in suggesting that a subset of the human population becomes increasingly infectious to mosquitoes at the onset of mosquito activity [1] and that the factor responsible may be the dramatic increase in mosquito bites themselves. Moreover, it should be noted that mosquito bites do not need to be from anophelines. Nuisance culicine mosquitoes are often found to be well in excess of anophelines [61-63] and notably were so in the Rosenberg study [59]. Mosquito spp. differ in their seasonal bionomics and some culicines (*Aedes *spp.), for example, have a capacity to increase more rapidly in numbers than anophelines; this is because of the ability of their eggs to resist dessication. Thus, the seed population of culicines is probably greater than that of anophelines, which must expand from pockets of aestivating females. Clearly this view is very preliminary, but the available information is encouraging and this hypothesis warrants closer examination in regions of endemic seasonal transmission.

If, indeed, there is a role for mosquito bites, this could have far reaching possibilities, not only for novel intervention strategies, but also for strategies in combatting malaria in regions of seasonally intense transmission, including regions at risk of epidemics. If intervention measures can be taken to reduce the potential reservoir of infection, by targeted use of antimalarial drugs with gametocytocidal effects during the window of mosquito resurgence, for example, the increase in transmission intensity will be severely delayed. Moreover, reducing human-mosquito contact by the use of insecticide treated bednets (ITBNs) or repellents may have considerable impact at this crucial time. A mass action community level benefit of ITBNs has already been noted, where individuals not sleeping under nets in villages where bednets were implemented, had reduced mortality and morbidity rates [64,65]. ITBNs can clearly have several community level beneficial effects and it would be of considerable interest to see if ITBNs actually alter the transmissibility of the parasite as well as reducing the burden of disease.

## Authors' contributions

RELP, MD and PTB were all involved in the conception of the article that arose out of lengthy discussions among the authors. The final draft was written by RELP with various sections added and/or removed by MD and PTB.

**Figure 4:**
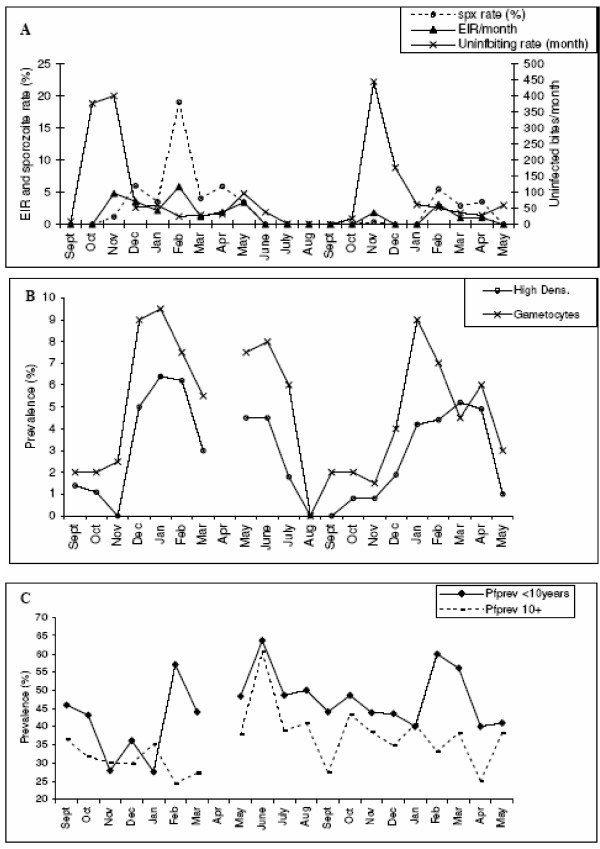
Longitudinal study of *P. falciparum *prevalence rates in a rural village in Thailand. Data from Rosenberg *et al *(1990) [58-60]. (a) Entomological parameters measured in sentinel houses. Number of infected (EIR) and uninfected mosquito bites per person per month and the sporozoite rate. (b) *P. falciparum *high trophozoite density and gametocyte prevalence rates. (c) Age-specific *P. falciparum *prevalence rates. Two age groups are considered: Greater than and less than 10 years old.
